# A Balanced Scorecard for Maximizing Data Performance

**DOI:** 10.3389/fdata.2022.821103

**Published:** 2022-03-31

**Authors:** Elizabeth Pierce

**Affiliations:** Department of Information Science, University of Arkansas at Little Rock, Little Rock, AR, United States

**Keywords:** data governance, monetization, data literacy, scorecard, metrics

## Abstract

A good performance monitoring system is crucial to knowing whether an organization's efforts are making their data capabilities better, the same, or worse. However, comprehensive performance measurements are costly. Organizations need to expend time, resources, and personnel to design the metrics, to gather evidence for the metrics, to assess the metrics' value, and to determine if any actions should be taken as a result of those metrics. Consequently organizations need to be strategic in selecting their portfolio of performance indicators for evaluating how well their data initiatives are producing value to the organization. This paper proposes a balanced scorecard approach to aid organizations in designing a set of meaningful and coordinated metrics for maximizing the potential of their data assets. This paper also discusses implementation challenges and the need for further research in this area.

## Introduction

Management and measurement are closely intertwined. Peter Drucker observed that organizations need feedback such as measurements to know whether they should stick to their current set of plans or whether it is time to change course and try something different (Zak, 2013). However, designing a measurement system to provide useful feedback is challenging. Redman (2005) noted that building a successful measurement system for data requires organizations to first identify the business requirements driving the need for measurement and how those metrics should be defined.

Moreover, when firms are trying to understand a situation as complex as how data is being managed and used to create value in their organization, the quantity and quality of metrics required to gain a full picture becomes even more daunting. Seiner (2016) described data governance as “the practice of applying formal accountability and behavior to assure the quality, effective use, compliance, security, and protection of data.” One need only consult several popular data governance frameworks to see the breadth of potentially measurable areas that data governance encompasses (Table 1).

**Table 1:** A sample of data governance frameworks and their major components.

**IBM** **(NASCIO, 2009)**	**DAMA** **(NASCIO, 2009)**	**Informatica (Guess, 2012)**
Data Risk Management and Compliance	Data Architecture Management	Policies
Value Creation	Data Development	Defined Processes
Organizational Structure and Awareness	Database Operations Management	Change Management
Policy	Data Security Management	Program Management
Stewardship	Reference and Master Data Management	Organizational Alignment
Data Quality Management	Data Warehousing and Business Intelligence Management	Dependent Processes
Information Life-Cycle Management	Document and Content Management	Measurement
Information Security and Privacy	Meta Data Management	People
Data Architecture	Data Quality Management	Vision
Classification and Metadata		Business Case
Audit Information Logging and Reporting		Tools and Architecture

Furthermore, data governance is only one aspect of what organizations should track about their data. Companies also want to know how best to monetize their data. In his book, Infonomics, Laney (2018, p. 29) lists a variety of ways for how organizations can use data for generating value.

1. Increasing customer acquisition/retention
2. Creating a supplemental revenue stream
3. Introducing a new line of business
4. Entering new markets
5. Enabling competitive differentiation
6. Bartering for goods and services
7. Bartering for favorable terms and conditions, and improved relationships
8. Defraying the costs of information management and analytics
9. Reducing maintenance costs, cost overruns, and delays
10. Identify and reducing fraud and risk
11. Cashing in on improved business performance
12. Improving citizen well-being.

Finally, there is growing realization that in order for organizations to achieve the full potential of their data, they need to invest in the data literacy of their workforce. According to Panetta (2021), Gartner defines data literacy as “the ability to read, write and communicate data in context, including an understanding of data sources and constructs, analytical methods and techniques applied, and the ability to describe the use case, application and resulting value.” Without employees possessing the necessary data, skills, tools, and motivation to take on projects that generate business value from data, organizations lack the environmental readiness needed for successful data monetization (Pigni et al., 2016).

Despite these complexities, organizations cannot afford to ignore measuring the performance of their data initiatives. The lack of a comprehensive data performance evaluation system means an impaired ability for organizations to manage their data assets which has serious consequences.

1. Difficulties retaining the Chief Data Officer. The chief data officer (CDO) is a senior executive responsible for the utilization and governance of data across the organization (Zetlin and Olavsrud, 2020). A recent survey by NewVantage Partners estimated that approximately 60% of organizations have hired a CDO (Bean, 2020). Despite the growing prominence of CDOs in corporate c-suites, many of these individuals are struggling to find success as evidenced by CDOs have one of the highest turnover rates among c-level executives with an average job tenure of ~2.5 years (DataKitchen, 2018). Bennett (2016) identified three major obstacles facing CDOs: (1) Organizational lack of understanding of the CDO role including a lack of focus in defining the most important initiatives; (2) Lack of stakeholder involvement and support along with a lack of resources and funding to support the CDO; and (3) Insufficient authority to execute responsibilities. A better system for coordinating data objectives, measures, and initiatives would help organizations to give their business leaders a clearer sense of expectations and responsibilities for data.
2. Difficulties maintaining compliance with data privacy and protection policies. Organizations need to understand who is using their data, how the data is being used and shared, and where the data is being stored (Brockman, 2020). Organizations need to have robust data governance in place to ensure that they have the policies, oversight, training, and systems in place to adequately protect their data and to ensure that their data complies with security and privacy regulations.
3. Difficulties reducing the operational costs of gathering, organizing, and sharing data. Better data governance has been shown to help reduce data duplication, strengthen data integration between applications, consolidate data storage, and improve data quality for faster, more reliable decision making (Sia Partners, 2020). Organizations need to evaluate their data governance efforts along with their data quality, data infrastructure, and data services to ensure their data operations are working at desired levels.

Articulating a clearer vision of critical data performance objectives is the first step to building a measurement system to support organizations' efforts to be more competitive with their data. To address this challenge, this paper proposes using a balanced scorecard management system that is specifically designed for maximizing the value of an organization's data assets. The rest of this paper is organized as follows. Section Balanced Scorecards gives a brief history of the use of balanced scorecards. Section Designing a Balanced Scorecard for Data Performance discusses what senior leaders should consider in designing a master balanced scorecard for data performance. Section Implementation Challenges and Closing Thoughts outlines the next steps necessary to implement the balanced scorecard approach throughout the organization along with plans for future research in this area.

## Balanced Scorecards

Kaplan and Norton (1992) developed the balanced scorecard (BSC) in the early 1990s as a management system that views an organization from several different perspectives in order to provide a more complete picture of performance. The balanced scorecard approach addressed the concern that organizations were placing too much emphasis on the results of financial performance, and not enough emphasis on the aspects of the business that lead to financial performance such as customer satisfaction and efficient business operations or the aspects of the business that lead to future financial performance such as learning and growth. The balance scorecard approach helps senior managers to think through their competing agendas to better identify the different strategies in play (ex. serving customers, reducing waste, increasing revenue) and how to work together to achieve results that benefit the organization as a whole and not just one area of the firm (Kaplan and Norton, 1992).

**Figure 1:**
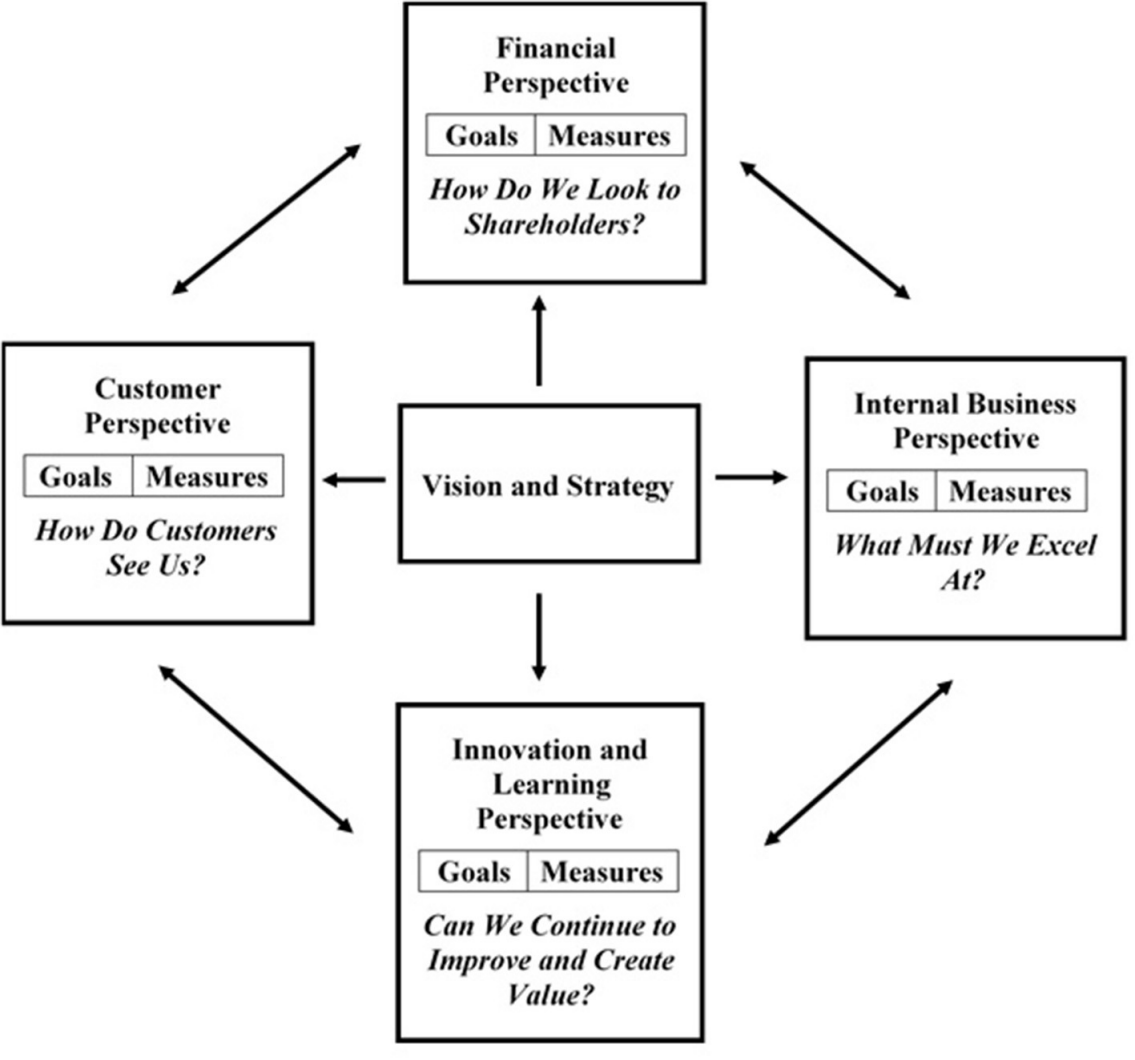
The balanced scorecard links performance measures (Kaplan and Norton, 1992).

Using the balanced scorecard management system (Figure 1), senior leaders choose the perspectives that best define the crucial areas necessary for sustaining business success. For each perspective, senior leaders identify a set of essential goals/objectives which further detail how the organization plans to achieve success. Each goal is associated with typically one or two key measures for evaluating how well the organization is reaching that goal. Further iterations of the scorecard include additional details such as the desired target for each metric, the current value for the metric, initiatives for improvement, and the person or group responsible for tracking that metric.

One of the main selling points of the balanced scorecard management system is that it is highly adaptable. The perspectives of the classic balanced scorecard can be changed to fit a wide variety of sectors including education, banking, airlines, manufacturing, healthcare, government, and non-profits. As a result, the balanced scorecard approach has been widely adopted by organizations. Hickman (2012) estimated that balanced scorecards are in use by 70% of companies around the world. There are also numerous software packages (both open source and vendor) available to support the implementation of a balanced scorecard management system.

Lastly, it is important to note that the balanced scorecard is a management system that requires intensive organizational commitment. In their study of Australian firms that had adopted the balanced scorecard approach, Chavan (2009) observed that to be successful, organizations had to overcome several obstacles. First, senior leadership had to reach a consensus as to what the master scorecard should look like for the organization. Next, the various business units needed to develop their unit scorecards whose objectives needed to align and contribute to the master scorecard. This process is then repeated at the department level and even at the individual level (e.g., employee performance appraisals) to ensure that all parties in the organization are working in integrated harmony to optimize business performance. Chavan (2009) concluded that organizations could achieve significant results using the balanced scorecard approach, but it requires leadership, communication, consensus, and accountability to be successful.

## Designing A Balanced Scorecard For Data Performance

In Laney's (2018, p. 1–4) book, Infonomics, he describes an infosavvy organization as one where business leaders can fully utilize information as a corporate asset, information is fully accessible to those who have a legitimate need, and the value of information assets can be measured. When it comes to using a balanced scorecard approach to assist organizations in achieving “infosavvy” status, the literature is sparse. A February 2022 literature review of Google Scholar using the search terms “scorecard” and “data” in the title yielded 122 results. The vast majority of these articles discussed various data issues with balanced scorecards such as improving performance assessment through techniques like data envelopment analysis, addressing data quality issues that impair the accuracy of performance metrics, and examining how big data systems could help automate the gathering of scorecard measures. Only four articles dealt with using balanced scorecards to improve data performance and those were in the context of assessing the success of a single data warehouse or similar large data store (Bensberg, 2003; Toomanian et al., 2011; Rahman, 2013; Martins and Belo, 2017). For organizations interested in a more ambitious balanced scorecard management system to help them maximize the performance across a wide portfolio of data assets, senior leaders must begin with articulating the perspectives, goals, and appropriate measures for deriving value from data that they want the rest of the organization to follow.

To help senior leaders define their data performance objectives, this paper draws its balanced scorecard inspiration from Pigni et al.'s (Pigni et al., 2016) article on generating value from Digital Data Streams. In their review of how firms innovate using digital data streams, Pigni et al. (2016) described five opportunities for how companies derive value from data along with four “readiness components” needed to create an environment conducive for pursuing those opportunities. The results of their analysis provide a sound basis for the perspectives of a balanced scorecard for guiding organizations on how to derive value from their data assets. Table 2 shows how the perspectives for the balanced data scorecard map to Pigni et al. (2016) Data Streams Value Framework. These data driven perspectives correlate well with the perspectives of the classic balanced scorecard: Data Monetization to Financial, Data Consumer to Customer, Data Governance to Internal Business, and Data Readiness to Innovation and Learning.

**Table 2:** Overview of data scorecard perspectives and their roles in generating data value.

**Balanced Data Scorecard Perspective**	**Pigni et al. (2016) Digital Data Streams Value Framework**
Data Monetization: Value generated when an organization is able to use data to generate financial or competitive value such as increasing revenue, reducing costs, or making better decisions.	Service—Value generated when an organization is able to use data and analytics to provide new services or to improve existing services. This value can take the form of either immediate action in response to a customer's service needs or insights as to how to compete more effectively. Efficiency—Value generated when an organization is able to use data and analytics to improve its business operations, better monitor its performance, or make its internal processes more efficient. This value can take the form of either immediate action such as emergency maintenance to replace a failing component or insights as to new ways to help employees be more productive. Analytics—Value generated when an organization is able to use data and analytics to gain understandings that help its executives and employees make better decisions. This value mainly takes the form of insights but is considered to be especially high value in that these insights could lead to new strategic directions that transform the organization.
Data Consumer—Value generated when an organization's personnel has quick and easy access to data that are defined, available, complete, organized, clean, and trustworthy.	Dataset Readiness Component—The ability to identify and access data that can generate value.
Data Governance—Value generated when data governance is conducted efficiently, effectively, equitably, and with good political acceptability.	Generation—Value generated from identifying the various sources of digital data available to organizations. These digital data assets could include social media data, machine to machine data, human generated data, transactional data, or biometric data. This archetype is preparatory in nature, meaning it lays the foundation for future strategic use of the data. Aggregation—Value generated when an organization collects, organizes, enhances, and repurposes data to create information services and platforms. This archetype is also preparatory in nature, meaning it lays the foundation for future strategic use of the data.
Data Readiness—Value generated when people have the data skills, access to data tools, and willingness to pursue data initiatives.	Skillset Readiness Component—The capability to orchestrate the skills necessary to deliver value from data. Toolset Readiness Component—The capability to use appropriate technology to realize value from data. Mindset Readiness Component—The organization's culture, strategy, and willingness to pursue data initiatives.

### Goals and Measures for Data Monetization (Financial Perspective)

There is widespread consensus that organizations need to achieve financial or competitive gains from their data (Goodwins, 2018; Laney, 2018, p. 28–52; Lonnon, 2018; Sakpal, 2019; Ahmad, 2020; Uttamchandani, 2020). For the Data Monetization perspective, senior leaders have several ways for how firms can derive value from their data assets.

1. Increase Revenue: Organizations can use data to increase their revenue streams. Examples of this include using data to aid in customer acquisition and retention efforts (Laney, 2018, p. 29–31; Sakpal, 2019; Earley, 2020), using data to improve sales or speed of delivery of products and services (Henderson, 2015; Laney, 2018, p. 35–37), developing new lines of business based on innovative usages of data (Laney, 2018, p. 33–34; Earley, 2020), or developing partnerships involving the sale or bartering of data to obtain more favorable terms or exchanges involving goods and services (Laney, 2018, p. 38–39).
2. Increase Market Presence: Organizations can use data to enhance the organization's stature through better corporate citizenship such as sponsoring open data portals or improving one's ranking in industry reports (Ajilitee, 2011; Laney, 2018, p. 45–48). While this may not have a direct impact on the firm's bottom line, it can boost an organization's brand recognition, adding to its social capital.
3. Reduce Costs: Organizations can use data to reduce its operating costs (Ajilitee, 2011; Henderson, 2015; Firican, 2018; Laney, 2018, p. 41–44; Foster, 2019; Stedman, 2020).
4. Improve Performance: Organizations can use data to help improve the performance (e.g., timeliness, quality) of its operations (Henderson, 2015; Subramanian, 2017; Laney, 2018, p. 36–37).
5. Improve Decision Making: Organizations can use data to enable their decision-makers to make decisions faster (Laney, 2018, p. 40–41), or with greater confidence (Foster, 2019), or with less chance of error (Subramanian, 2017).
6. Reduce Risk: Organizations can use data to reduce risk such as being able to better spot fraud, security breaches, or privacy issues which improves the bottom line through improved regulatory compliance and avoidance of penalties (Ajilitee, 2011; Smith, 2017; Subramanian, 2017; Laney, 2018, p. 44–45).

Table 3 summarizes some examples of goals and metrics related to data monetization. Organizations should adjust the emphasis placed on the various options depending on the strategic priorities of the firm.

**Table 3:** Data monetization example goals and metrics.

Data Monetization: Value generated when an organization is able to use data to generate financial or competitive value such as increasing revenue, reducing costs, or making better decisions.	
**Example Goals**	**Example Metrics**
Increase Revenue	Number of new lines of business spawned by new forms of data usage bringing innovative solutions/services to the market (Laney, 2018; Earley, 2020) Increased customer acquisition and retention such as data initiatives resulting in percentage reduction in shopping cart abandonment (Laney, 2018)
Increase Market Presence	Increased customer interest such as data initiatives resulting in percentage increase in attendance at events Laney (2018): Improved standing in industry report cards due to better data (Ajilitee, 2011)
Reduce Costs	Amount of operational costs reduced after implementing data improvements (Firican, 2018; Laney, 2018) Reduction in number of delays due to data integrity issues (Foster, 2019)
Improve Performance	Reduction in client complaints due to delayed or inaccurate billing (Henderson, 2015; Subramanian, 2017) Reduction in time to complete tasks due to data improvements (Henderson, 2015)
Improve Decision Making	Reduction in time required for making data driven decisions (Laney, 2018) Reduction in number of inaccurate decisions due to bad data (Subramanian, 2017)
Reduce Risk	Reduction in the number or cost of regulatory fines/penalties (Ajilitee, 2011; Smith, 2017; Subramanian, 2017; Laney, 2018) Reduction in the number or cost of security breaches and/or fraud losses (Ajilitee, 2011; Firican, 2018; Seiner, 2018; Foster, 2019; Stedman, 2020)

### Goals and Measures for Data Consumer (Customer Perspective)

Getting the right data to the organization's constituents who need to work with that data is another priority for organizations to fully realize the value of their data (Dyché J., 2018; Sakpal, 2019; Ahmad, 2020; Uttamchandani, 2020). For the Data Consumer perspective, keeping data consumers satisfied requires that the organizations deliver on several significant areas.

1. Ensure Data Quality: Making sure that data is fit for use is a widely cited goal among industry practitioners. Organizations need to make sure that their data is valid, consistent, current, and complete for a given data source (Ajilitee, 2011; Loshin, 2013; Henderson, 2015; Dennis, 2017; Subramanian, 2017; Firican, 2018; NYU Langone Health System, 2018; Seiner, 2018; Foster, 2019; Stedman, 2020) as well as between related data sources (Ajilitee, 2011; Henderson, 2015; Dennis, 2017; Stedman, 2020).
2. Build Data Infrastructure: Organizations need to make sure there is an adequate infrastructure in place that allows data consumers to easily find and access the data that they need. Designing an efficient and secure data ecosystem that supplies data where needed is another important performance area for organizations (Ajilitee, 2011; Henderson, 2015; Dennis, 2017; Smith, 2017; Firican, 2018; Laney, 2018, p. 80–102; Earley, 2020).
3. Deliver Data Services: Data consumers in the organization require services such as a data helpdesk so they can get their access requests, questions, and concerns about their data addressed. Key areas for measuring data service quality include the variety of data identified and available to data consumers (Ajilitee, 2011; Henderson, 2015; Dennis, 2017; Seiner, 2018; Stedman, 2020), as well as the satisfaction of the data consumers with the timeliness, reliability, and completeness of data services provided (Ajilitee, 2011; Loshin, 2013; Henderson, 2015; Dennis, 2017; NYU Langone Health System, 2018; Stedman, 2020).

Table 4 summarizes some examples of goals and metrics related to satisfying data consumers. Organizations should adjust the emphasis placed on the various options depending on the strategic priorities of the firm.

**Table 4:** Data consumer example goals and metrics.

Data Consumer: Value generated when an organization's personnel has quick and easy access to data that are defined, available, complete, organized, clean, and trustworthy.	
**Example Goals**	**Examples Metrics**
Ensure Data Quality	Percentage of data attributes that meet desired quality dimensions such as completeness, correct format, valid values, non-duplicates, or currency (Ajilitee, 2011; Loshin, 2013; Henderson, 2015; Dennis, 2017; Subramanian, 2017; Firican, 2018; NYU Langone Health System, 2018; Foster, 2019; Stedman, 2020) Increase in customer satisfaction with areas such as the trustworthiness of data, the availability of data, conformance of data to industry standards, or the extent to which data is easy to understand, manipulate, and apply to different tasks (Ajilitee, 2011; Seiner, 2018; Stedman, 2020).
Build Data Infrastructure	Number of consolidated, integrated and shared master data sources (Henderson, 2015; Dennis, 2017; Smith, 2017; Firican, 2018) Increased speed in delivering data (Laney, 2018, p. 80-102),
Deliver Data Services	Adherence to terms specified in Service Level Agreements (Ajilitee, 2011; Loshin, 2013) Time to complete data tickets such as response to data-related inquiries from business users, improvement requests for common data entities, attribute changes, data enrichment requests, access requests, or other requested data updates (Dennis, 2017; NYU Langone Health System, 2018; Stedman, 2020).

### Goals and Measures for Data Governance (Internal Business Perspective)

Good data governance is essential to an organization's ability to work with its data. Dennis (2017) identifies three fundamental areas: people, processes, and technology that must be addressed for effective data governance.

1. Engage People: For data governance to be effective, it is important that there is engagement among the different stakeholders. Organizations need to pay attention to the level of interactions between their data governance council and their business process owners, data stewards, IT personnel, and data consumers regarding their awareness of data governance policies and practices, their willingness to provide input to data governance deliberations, and their commitment in following through on desired data behaviors (Ajilitee, 2011; Loshin, 2013; Henderson, 2015; Dennis, 2017; Smith, 2017; Firican, 2018; Seiner, 2018; Stedman, 2020).
2. Get Data Processes Right: Organization need to make certain that data governance processes (including those for generating and enforcing data policies) are streamlined and responsive to the needs of the organization and its employees. Organizations need to monitor the extent to which data policies and processes have been developed and adopted by their personnel (Henderson, 2015; Dennis, 2017; Smith, 2017; Seiner, 2018; Stedman, 2020). Organizations should also track improvements to data policies and processes that resulted in elimination of duplicate effort, reduction of data errors or failures, or improved regulatory compliance (Ajilitee, 2011; Henderson, 2015; Dennis, 2017; Subramanian, 2017; Firican, 2018; Seiner, 2018; Foster, 2019; Stedman, 2020).
3. Automation of Effort: As the amount of data grows, technology is required to help automate tasks to make it easier and quicker for an organization's data governance efforts to keep up. Organizations should consider metrics that track the reduction in manual effort (Ajilitee, 2011; Loshin, 2013; Henderson, 2015; Dennis, 2017; NYU Langone Health System, 2018; Stedman, 2020) along with metrics that track the time savings (Ajilitee, 2011; Loshin, 2013; Henderson, 2015; Dennis, 2017) associated with automation improvements.

Table 5 summarizes some examples of goals and metrics related to delivering effective data governance. Organizations should adjust the emphasis placed on the various options depending on the strategic priorities of the firm.

**Table 5:** Data governance example goals and metrics.

Data Governance: Value generated when data governance is conducted efficiently, effectively, equitably, and with good political acceptability.	
**Example Goals**	**Examples Metrics**
Engage People	Number of data issues/projects taken up by the Data Governance Council along with their status (e.g., number of data issues/projects approved, in the backlog, in progress, successfully completed) along with who participated and their time spent from issue identification to issue resolution (Ajilitee, 2011; Loshin, 2013; Henderson, 2015; Dennis, 2017; Seiner, 2018; Stedman, 2020) Number of data owners/stewards identified vs. number of domains (Henderson, 2015; Dennis, 2017)
Get Data Processes Right	Percentage or number of business processes that utilize common data definitions/standards, by subject area (Dennis, 2017; Smith, 2017), Results of compliance tests or audits reporting on the extent to which access to data is restricted appropriately to maintain its security, timely deletion of sensitive data, data access rights management, or the number of regulatory non-compliance data issues with HIPAA, PHI or other policies (Ajilitee, 2011; Henderson, 2015; Firican, 2018; Seiner, 2018; Foster, 2019; Stedman, 2020)
Automation of Effort	Reduction in cost due to greater efficiencies such as reducing the number of times data processes require manual intervention such as match-merge logic (Ajilitee, 2011) Reduction in time such as time to produce a report (Dennis, 2017), to diagnosis and correct a data issue (Loshin, 2013), or to capture and deliver data (e.g., compliance data, log data, or system data) (Ajilitee, 2011; Henderson, 2015)

### Goals and Measures for Data Readiness (Learning and Growth Perspective)

The Data Readiness perspective of the balanced scorecard covers the aspects of the organization's environment that must be in place to encourage and enable employees to find new ways to innovate using data. Employees need to have the skills, tools, and willingness to pursue data analytics (Pigni et al., 2016; Sakpal, 2019; Ahmad, 2020; Uttamchandani, 2020). Without these components, organizations may find that they have created a large data engine for their organization without the necessary enablers for employees to make use of that engine (Pigni et al., 2016; Bean, 2021). In order to facilitate a culture of data literacy, organizations need to work on the following important areas.

1. Increased Skills Training: In order to analyze data, employees need to have the prerequisite knowledge and skills. Organizations should periodically assess the level of skills in the organization's employees and to identify areas for improvement (Dennis, 2017; Firican, 2018; Seiner, 2018; Sakpal, 2019; Ahmad, 2020; Uttamchandani, 2020).
2. Access to Data Tools: In addition to skills, employees need access to a rich set of tools for querying, wrangling, and analyzing their data (Earley, 2020; Uttamchandani, 2020).
3. Grow an Inquiring Mindset: Finally employees need to be encouraged and rewarded for participating in data initiatives (Ajilitee, 2011; Sakpal, 2019; Ahmad, 2020; Uttamchandani, 2020). For organizations, this means using a variety of tactics such as newsletters, bonuses, annual evaluations, and surveys to recognize individuals who are innovating and developing solutions with data.

Table 6 summarizes some examples of goals and metrics related to fostering data readiness. Organizations should adjust the emphasis placed on the various options depending on the strategic priorities of the firm.

**Table 6:** Data readiness example goals and metrics.

Data Readiness: Value generated when people have the data skills, access to data tools, and willingness to pursue data initiatives.	
**Example Goals**	**Example Metrics**
Increase Skills Training	Number or percentage of people who have received training (either through the on-boarding of people, professional development, or through participation in other tasks and activities related to growing the data analytical knowledge and skills of employees (Dennis, 2017; Seiner, 2018) Number or percentage of surveyed employees indicating what data skills they possess along with their satisfaction level with their skills (Firican, 2018; Sakpal, 2019; Ahmad, 2020; Uttamchandani, 2020)
Access to Data Tools	List of available data/analytical tools along with the resources (e.g., training and support) for using these tools (Earley, 2020; Uttamchandani, 2020) Number or percentage of surveyed employees indicating their satisfaction level regarding their training, access, and usage of available analytical tools (Uttamchandani, 2020)
Grow an Inquiring Mindset	Number or percentage of surveyed employees indicating their involvement with data projects (Ajilitee, 2011; Sakpal, 2019; Uttamchandani, 2020), Number of employees receiving awards or recognitions for using data in innovative ways (Sakpal, 2019; Ahmad, 2020)

## Implementation Challenges And Closing Thoughts

**Figure 2:**
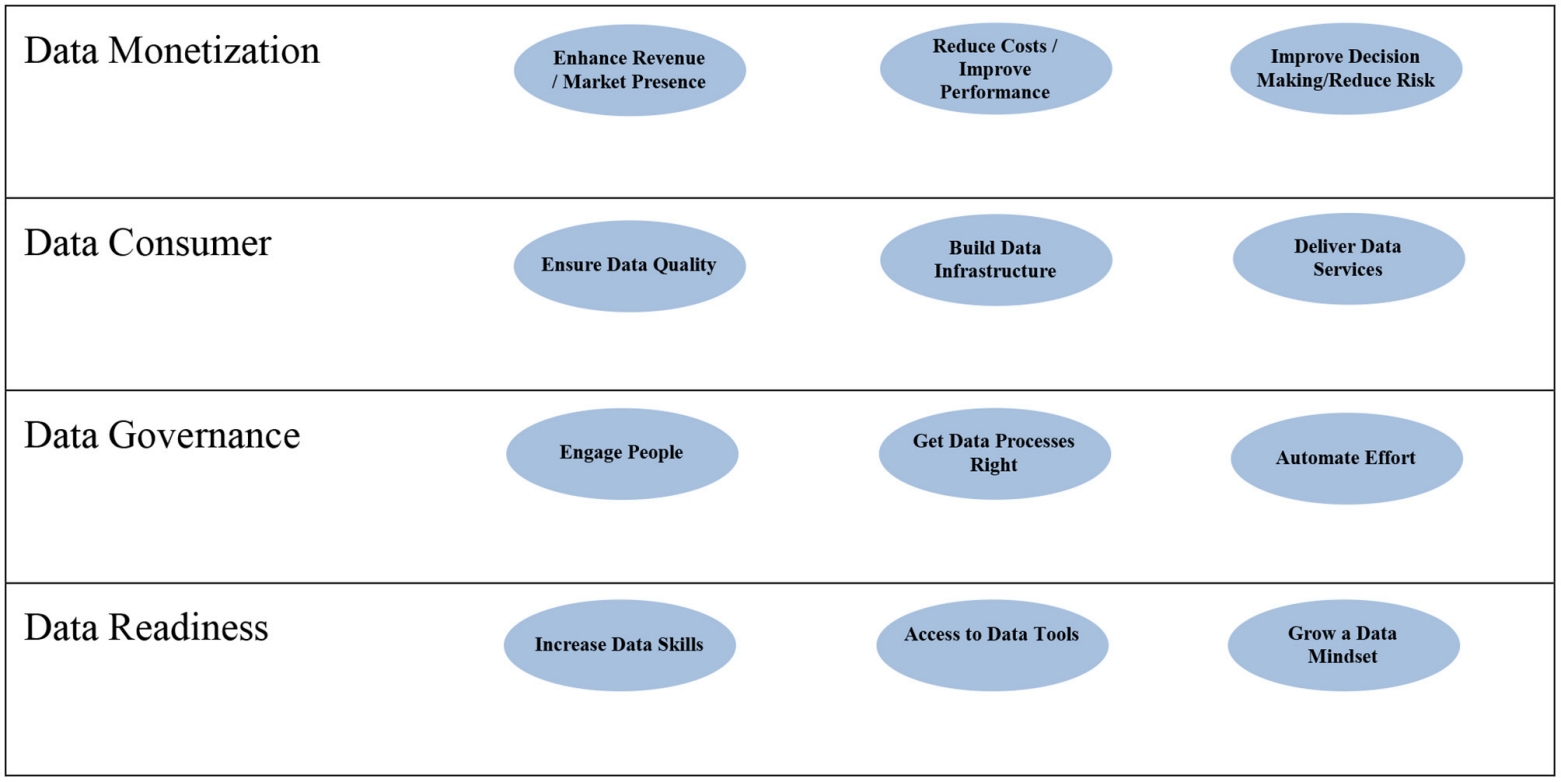
Key perspective and goals for the balanced data scorecard.

The balanced data scorecard is a starting point for senior leaders on how they would like their organization to derive value from data. In addition to identifying data monetization objectives, the balanced data scorecard also helps organizations to identify their data consumers' needs, data governance efforts, and the data readiness that must be in place to make it possible for that value to be generated. Figure 2 provides an overview of the main perspectives and goals presented in this paper. Organizations are free to adapt these perspectives and goals to meet their specific circumstances.

The master balanced data scorecard generated by senior leadership is the beginning of a long process. The next steps involve the various divisions and departments of the organization generating their own versions of the balanced data scorecard to support the master data scorecard. These scorecards must be shared and vetted to ensure that the collection of balanced data scorecards work together as a whole without creating suboptimal situations where one group's pursuit of their data objectives undermine the overall efforts of other groups in the organization. Once a portfolio of balanced data scorecards has been established then the organization must follow through with a regular cycle of gathering the metrics for each of the performance goals, assessing the current state of data, devising action plans for improvement for the coming cycle, and updating the various balanced data scorecards to keep them relevant to the needs of the firm.

Organizations will need a comprehensive data measurement system to accompany their balanced data scorecard management system. While the balanced data scorecard will answer some questions regarding the business needs that are driving measurement and how those metrics should be defined, Redman (2005) lists a number of other important questions that must be addressed for successful performance monitoring to occur.

- Where and when should the measurements be taken?
- Who is responsible for producing the metric?
- What measurement device or protocol should be used?
- What data (evidence) to include in the calculation of the metric?
- How should results be summarized and reported?
- How should the measurements be interpreted and used for taking action?

While it would be great if there was a shorter path for organizations to attain the full potential of their data, the hard truth is data is a complicated asset. It is generated, managed, and used throughout the organization. Consequently, data requires the approach of the balanced data scorecard management system to get all parties working together to plan, collect, store, share, and manage data so it can be monetized to create significant value for the organization. More work is needed to study the usefulness of the balanced data scorecard for assisting organizations in improving their bottom lines. At the very least, the balanced data scorecard is a framework for senior leaders to consider when strategizing how their organization can more effectively capitalize upon their data assets.

## Author Contributions

The author confirms being the sole contributor of this work and has approved it for publication.

## Funding

This material is based upon work supported by the National Science Foundation under Award No. OIA-1946391.

## Conflict of Interest

The author declares that the research was conducted in the absence of any commercial or financial relationships that could be construed as a potential conflict of interest.

## Publisher's Note

All claims expressed in this article are solely those of the authors and do not necessarily represent those of their affiliated organizations, or those of the publisher, the editors and the reviewers. Any product that may be evaluated in this article, or claim that may be made by its manufacturer, is not guaranteed or endorsed by the publisher.
